# Shoulder Prosthetic Infection and Humerus Osteomyelitis From Cutibacterium Following Eden-Hybinette Procedure

**DOI:** 10.7759/cureus.56289

**Published:** 2024-03-16

**Authors:** Enrico M Zardi, Alessio Palumbo, Edoardo Giovannetti De Sanctis, Francesco Franceschi

**Affiliations:** 1 Medicine and Surgery, Fondazione Policlinico Universitario Campus Bio-Medico, Rome, ITA; 2 Orthopaedic and Trauma Surgery, Ospedale San Pietro Fatebenefratelli, Rome, ITA; 3 Orthopaedic and Trauma Surgery, Saint Camillus International University of Health Sciences (UniCamillus), Rome, ITA

**Keywords:** therapy, osteomyelitis, surgical site infection, shoulder dysfunction, cutibacterium acnes

## Abstract

We describe the case of a patient who recently underwent a guided arthroscopic Eden-Hybinette procedure for the revision of a previous failed procedure of Latarjet and resurgence of shoulder instability. The subsequent development of painful infectious arthritis of the left shoulder complicated by osteomyelitis of the humerus, caused by *Cutibacterium acnes*, and accompanied by high fever was resolved only after the removal of synthetic screws and bone grafting and thanks to prolonged intravenous antibiotic treatment. The antibiotic regime was continued, both intramuscularly and orally, after discharge, allowing the full healing of the severe osteoarticular infection of the shoulder.

## Introduction

*Cutibacterium acnes* (formerly *Propionibacterium acnes*) is a slow-growing gram-positive non-spore-forming anaerobic bacterium also known as commensal of the skin that, in some circumstances, may become an opportunistic pathogen, causing superficial and deep infections after surgical procedures and device implantation [1,2]. It is one of the most common bacteria causing prosthetic joint infection of the shoulder [3].

Shoulder joint instability is strongly dependent on changes in bone morphology of the glenoid and humeral head structures [4]. Among the best techniques to deal with shoulder instability are the arthroscopic Latarjet procedures [5] and, in case of their failure, the Eden-Hybinette procedures are frequently used [6].

Here, we describe a case of infectious shoulder arthritis complicated by osteomyelitis of the humerus, which arose a month after the Eden-Hybinette procedure performed for the resurgence of shoulder instability.

## Case presentation

**Figure 1:**
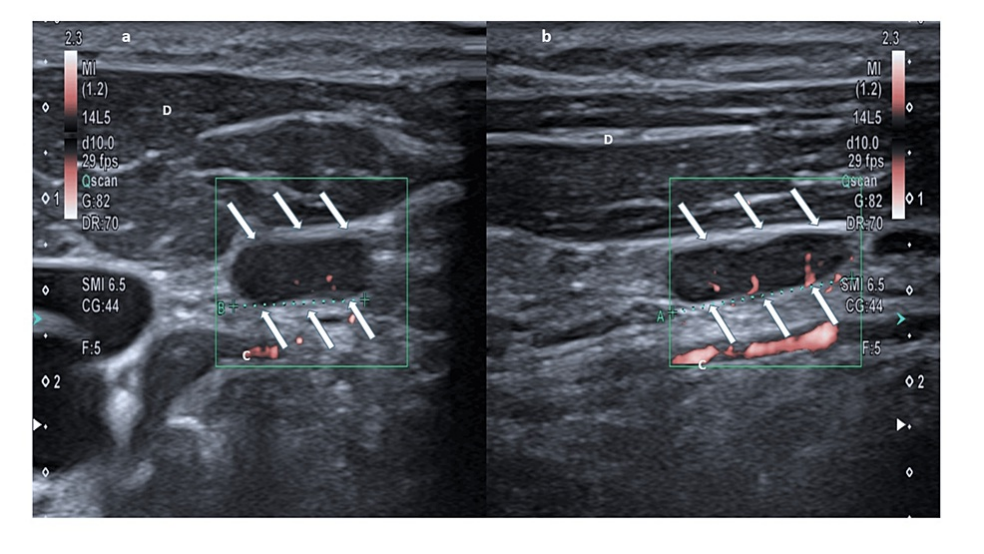
An enlarged and reactive lymph node (arrows) of the left shoulder in axial view (a) and longitudinal view (b). D: Deltoid muscle. C: Cephalic vein.

A 42-year-old man, about three years before, had performed a Latarjet revision using a bone coracoid block for his left shoulder instability [7]. Unfortunately, due to coracoid bone block fragmentation, following an accidental fall, he newly developed left shoulder instability. To resolve this problem, a guided arthroscopic Eden-Hybinette procedure, a technique allowing for an effective shoulder salvage after a failed Latarjet revision, was attempted, using a synthetic bone graft [8]. One month after the procedure, the patient had a light fever, shoulder pain, and an increase in C-reactive protein (1.8 mg/dl, normal value <0.5 mg/dl). Antibiotic therapy based on amoxicillin/clavulanate 875/125 mg twice a day was started without benefit. On suspicion of bone block graft rejection, a cortisone-based therapy (25 mg prednisone once a day with a dose reduction of 5 mg every 10 days until complete interruption) was administered for two months. However, the patient's clinical condition further worsened with shoulder pain and swelling. Therefore, an open surgery procedure was carried out to remove synthetic bone graft and screws, performing curettage and debridement of the glenoid surface; then, he was discharged with teicoplanin 200 mg i.m. twice a day. One week after discharge, he had an episodic appearance of chills followed by fever and was newly admitted to our hospital. Physical examination revealed asthenia, high fever (40.5°C), tachycardia (110 beats per minute), low artery pressure (95/60 mmHg) with swelling and severe pain of the left shoulder but normal transcutaneous monitoring of oxygen (97%) and upper and lower respiratory tract examination, normal auscultation of heart sounds, absence of heart murmurs and cardiac signs of failure, normal abdomen inspection, percussion, palpation, and auscultation. Blood analysis showed the following: C-reactive protein 3.5 mg/dl (normal value <0.5 mg/dl), D-dimer 2580 ng/ml (normal value <500 ng/ml), erythrocyte sedimentation rate 91 mm/h (normal value <17 mm/h), white blood cell count 8120 cells/µl, neutrophil count 4500 cells/µl (55%), red blood cell count 5,260,000 cells/µl, hemoglobin 13.8 g/dl, platelet count 350,000/µl, and creatinine 0.86 mg/dl. Urinary and blood cultures were negative. Thorax X-ray and echocardiography excluded pneumonia, endocarditis, and pericarditis, respectively. Abdominal sonography only showed a spleen area of 48 cm^2^ (normal values up to 45 cm^2^). Neck sonography showed multiple enlarged lymph nodes in the left lymphatic stations (Figure 1).

**Figure 2:**
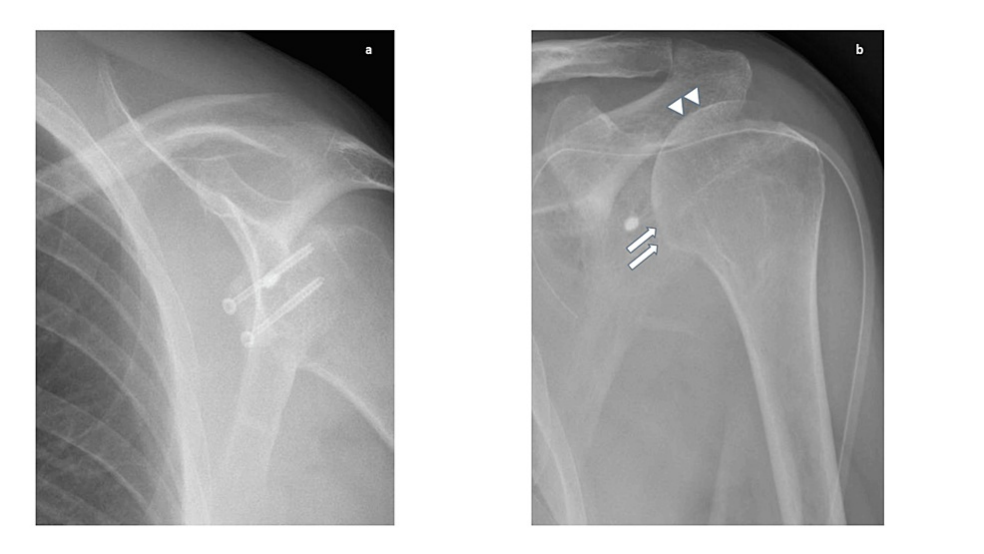
Anteroposterior shoulder radiograph before (a) and after (b) the removal of the compression screws. (b) Humeral head erosion (arrows) and thickening of the cortical bone (arrowheads).

The shoulder X-ray showed some erosion signs of the humeral head and the presence of synthetic screws (Figure 2).

**Figure 3:**
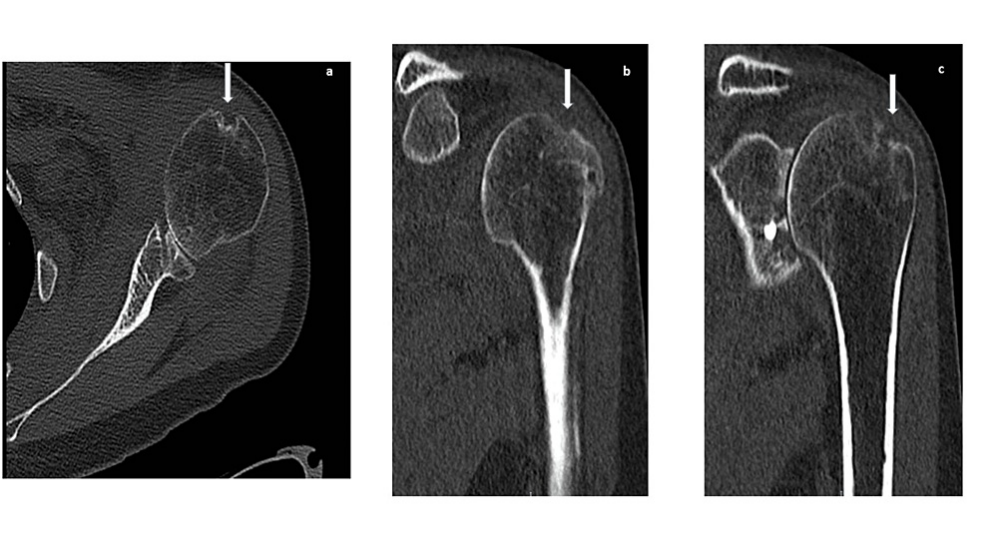
Shoulder computed tomography in axial view (a) and coronal view (b, c) after the removal of the screw. In all panels, osseous erosion and destruction of the cortical bone of the humeral head are present (arrows).

Computed tomography of the left shoulder revealed osteomyelitis signs (Figure 3).

Culture collection from the synthetic screws of the humerus, shoulder synovia and synovial fluid, and bone humerus were all positive for *Cutibacterium acnes*. Antibiotic treatment was quickly increased giving intravenous teicoplanin 400 mg twice a day, levoxacin 750 mg once a day, and ceftazidime 2 g twice a day. After two weeks of this full treatment regimen, the patient was discharged without fever with the following 30-day antibiotic prescription (intramuscular teicoplanin 200 mg twice a day and oral rifampin 600 mg once a day two hours after meals). Fifteen days after discharge, he was in good clinical condition, and his blood analysis showed the following: C-reactive protein 1.2 mg/dl (normal value <0.5 mg/dl), D-dimer 1509 ng/ml (normal value <500 ng/ml), erythrocyte sedimentation rate 43 mm/h (normal value <25 mm/h), white blood cell count 4460 cells/µl, neutrophil count 2520 cells/µl (55%), red blood cell count 5,080,000 cells/µl, hemoglobin 12.9 g/dl, platelet count 272,000/µl, and creatinine 0.78 mg/dl. Normalization of inflammatory markers was obtained after two more months.

## Discussion

*C. acnes* is considered a contaminating bacterium from the normal skin microbiota of patients, but it is also recognized as a bacterium able to cause true polyclonal infections [9]. The concomitant and indiscriminate use of topical agents (retinoids, benzoyl peroxide) makes it much more difficult to find the right and appropriate antibiotic treatment for acne. This, in turn, can lead to select bacteria resisting common antibiotics which may be the reason why this bacterium can become so insidious.

Interestingly, between 9% and 41% of patients present positive *C. acnes* culture in synovial fluid collected during primary shoulder arthroplasty [10,11]. It is known that isolation of *C. acnes* in joint and bone culture samples is difficult to decipher, but diagnosis is reached when, following the guidelines criteria, greater than (>) or equal to (=) two positive culture samples are present [12] or/and an increase of inflammatory markers [13] and a quick response to therapy are obtained: this happened in our case. 

*C. acnes* is therefore involved in up to 20% of all infections after shoulder arthroplasty [14]. In some favorable circumstances, it may be responsible for serious life-threatening infections. This case would prove that it does. Indeed, in our patient, there was no effective response to the first antibiotic regimen (intramuscular teicoplanin). This was also favored by a previous cortisone-based therapy started on the erroneous suspicion of an immunologic rejection against the synthetic bone graft in the glenoid surface of the shoulder. Only a full therapeutic regimen with three intravenous antibiotics allowed the fever disappearance, the shoulder pain reduction, the decrease of inflammatory markers, and the improvement of symptoms until full recovery.

## Conclusions

Although other reports have described the involvement of *C. acnes* in causing osteomyelitis, this is the first case described after an Eden-Hybinette procedure resolved with total recovery. *Cutibacterium* can be responsible for the onset of acute and severe febrile disease and osteomyelitis in patients who have had more than one surgical procedure on the same joint site and also underwent cortisone therapy. The acute phase can be resolved with a combination of parenteral antibiotics for at least two weeks, but, in order to avoid the resurgence of infection, continuation of therapy at home, until the disappearance of the main signs of inflammation, is necessary.
